# Comparison of Adaptive Behavior Measures for Children with HFASDs

**DOI:** 10.1155/2013/415989

**Published:** 2013-05-30

**Authors:** Christopher Lopata, Rachael A. Smith, Martin A. Volker, Marcus L. Thomeer, Gloria K. Lee, Christin A. McDonald

**Affiliations:** ^1^Institute for Autism Research, Canisius College, 2001 Main Street, Buffalo, NY 14208, USA; ^2^Department of Counseling, School and Educational Psychology, University at Buffalo, State University of New York, 409 Baldy Hall, Buffalo, NY 14260-1000, USA; ^3^Nationwide Children's Hospital, 187 W. Schrock Road, Westerville, OH 43081, USA

## Abstract

Adaptive behavior rating scales are frequently used to gather information on the adaptive functioning of children with high-functioning autism spectrum disorders (HFASDs), yet little is known about the extent to which these measures yield comparable results. This study was conducted to (a) document the parent-rated VABS-II, BASC-2, and ABAS-II adaptive behavior profiles of 6- to 11-year-olds with HFASDs (including relative strengths and weaknesses); (b) examine the extent to which these measures yielded similar scores on comparable scales; and (c) assess potential discrepancies between cognitive ability and adaptive behavior across the measures. All three adaptive measures revealed significant deficits overall for the sample, with the VABS-II and ABAS-II indicating relative weaknesses in social skills and strengths in academic-related skills. Cross-measure comparisons indicated significant differences in the absolute magnitude of scores. In general, the VABS-II yielded significantly higher scores than the BASC-2 and ABAS-II. However, the VABS-II and ABAS-II yielded scores that did not significantly differ for adaptive social skills which is a critical area to assess for children with HFASDs. Results also indicated significant discrepancies between the children's average IQ score and their scores on the adaptive domains and composites of the three adaptive measures.

## 1. Introduction

Children with high-functioning autism spectrum disorders (HFASDs; i.e., Asperger's disorder, autism (high-functioning), and PDD-NOS) share core diagnostic features including impairments in social relatedness and interactions and restricted and repetitive behaviors, interests, and activities [1]. They are considered high-functioning due to relative strengths in cognitive and formal language abilities, yet pragmatic communication deficits are common. Although these features characterize the diagnostic parameters of the disorders, they do not convey the degree of impairment in daily functioning. Klin et al. [2] characterized diagnostic symptomatology as indicative of *disability* and adaptive functioning as indicative of *ability* (strengths and weakness in daily functioning). Assessment of adaptive functioning is a necessary component of comprehensive evaluations for children with HFASDs [3, 4], as it provides critical information that assists with diagnosis, attainment of services, and treatment/educational programming [5, 6]. 

Adaptive functioning represents the individual's “ability to translate capacities into consistent, habitual behaviors fostering self-sufficiency in naturalistic settings” [7, page 775]. Examples of adaptive behaviors include communication, socialization, and self-care skills [5]. While studies have documented adaptive behavior levels in lower-functioning individuals with ASDs that parallel or exceed their cognitive levels (e.g., [8]), significant adaptive deficits have also begun to be reported in more cognitively capable individuals with HFASDs. A unique aspect of individuals with HFASDs is that their adaptive behaviors most often fall below their cognitive ability level [9]. In addition, the discrepancy between their adaptive behavior and cognitive ability level has been found to increase with age, suggesting that adaptive behavior does not keep the expected pace with chronological age or cognitive ability [10]. To date, many of the adaptive behavior findings related to HFASDs have come from studies utilizing heterogeneous samples (i.e., both lower- and higher-functioning individuals with ASDs; e.g., Kanne et al. [10]; Liss et al. [11]; Perry et al. [5]), with far fewer rigorously controlled studies focusing specifically on individuals with HFASDs.

In general, the studies that have focused on individuals with HFASDs have reported significant adaptive behavior deficits. In a review of eight earlier studies of adaptive behavior in individuals with Asperger's disorder (spanning 1995–2005), Lee and Park [12] found ratings were generally 1 SD to >2 SD below the population mean despite the average IQ of each sample falling in the average range. The greatest adaptive impairments were in the areas of socialization and daily living skills, with communication impaired but to a lesser degree. 

At present, the majority of what has been documented about the adaptive behaviors of individuals with ASDs/HFASDs has been derived using the Vineland Adaptive Behavior Scales (VABS; [13]). This scale is the most commonly used and studied measure of adaptive functioning for ASDs [2]. This was illustrated in the review by Lee and Park [12] that revealed that seven of the eight studies had used the VABS. 

Two more recent studies used the VABS to examine the adaptive behaviors of children and adolescents specifically with HFASDs. Klin et al. [2] assessed the adaptive behaviors of 7- to 18-year-olds with HFASDs (Verbal IQ (VIQ) > 70) across two research sites. While one site's sample was relatively more impaired, results indicated significant adaptive behavior deficits at each site and VABS standard scores from >1 SD to >3 SD below the population mean. Additionally, VABS scores were significantly discrepant from the samples' verbal ability scores (adaptive scores ranging from >1.5 SD to >3 SD below VIQ). For the specific adaptive areas, the pattern was consistent across sites with the Socialization domain most significantly impaired, followed by Daily Living Skills, and finally Communication. In a similar study using the VABS, Saulnier and Klin [14] evaluated adaptive behaviors (Socialization and Communication) in subsamples of 7- to 18-year-olds with Asperger's disorder and high-functioning autism. Results indicated that both groups exhibited significant Socialization, and to a lesser extent Communication, deficits compared to general population means (>−1.5 SD to >−3 SD), as well as relative to the participants' Full Scale and VIQs. While these studies illustrate the adaptive behavior deficits of individuals with HFASDs and common use of the VABS, an updated version of this measure (VABS-II; [15]) has been published including a survey (rating scale) version. As a result, additional studies of adaptive functioning in ASDs using the VABS-II are needed [5].

Given the extensive reliance on the original VABS, far less is known about adaptive behaviors in ASDs/HFASDs using other adaptive behavior measures. Kenworthy et al. [16] assessed the adaptive behaviors of 12- to 22-year-olds with HFASDs (*n* = 40) compared to matched controls (*n* = 30) using the Adaptive Behavior Assessment System-Second Edition (ABAS-II; [17]). Findings revealed significant deficits for the HFASD group in overall adaptive behavior, as well as on the three ABAS-II composites (i.e., Social, Conceptual, and Practical) relative to controls. All nine ABAS-II skill areas were significantly lower for the HFASD group than controls, with the social skills area most severely impaired. Further, composite scores for the HFASD group were generally ≥1.5 SD below the general population mean and fell farther below the group's IQ level (IQ M = 111.75). The authors concluded that the ABAS-II effectively documented adaptive functioning in this sample with HFASDs. However, they identified the study's primary limitation as the “failure to collect VABS data along with the ABAS data for the purposes of directly comparing the two measures in the same population” (page 421). The authors recommended that future studies directly compare these two measures for samples with ASDs as well as extend the research by using samples of children with ASDs/HFASDs.

Another exception was a recent study that utilized a broad measure of clinical and adaptive functioning for youth with HFASDs. Volker et al. [18] examined the clinical and adaptive features of 6- to 16-year-olds with HFASDs (*n* = 62) compared to matched controls (*n* = 62) using the Behavior Assessment System for Children-Second Edition, Parent Rating Scales (BASC-2 PRS; [19]). The Adaptive Skills Composite of the HFASD group fell significantly below the matched controls and more than 1.5 SD below the population mean. Similar deficits were noted for the HFASD group compared to matched controls for all five of the adaptive subscales. Compared with the BASC-2 PRS normative mean, subscale scores were approximately 1.5 SD below the population estimates and fell in the middle of the at-risk range. Volker et al. [18] concluded that the BASC-2 PRS was effective in detecting important features of children with HFASDs including adaptive behavior impairments. However, the authors suggested that additional studies are needed to evaluate the extent to which different assessment measures yield similar results regarding the severity of impairment in the target constructs.

While studies have begun to examine the adaptive behaviors of this population, there continues to be a need for studies that characterize the adaptive skill levels of individuals with HFASDs [2]. Because little is known about the adaptive behaviors of individuals with HFASDs of differing ages [2], and including individuals from a wide age range can obscure important age-related features [20], there is a need to study adaptive behaviors in more narrowly defined (i.e., age and IQ) groups [8]. Understanding of the adaptive functioning of these children is influenced by the skills assessed within a specific measure [11], yet no studies were identified that have documented and compared the adaptive skills of children with HFASDs using multiple measures within the same sample. Kenworthy et al. [16] and Volker et al. [18] emphasized the need for studies that address this significant gap. Primary aims of the current study were to (a) document the parent-rated VABS-II, BASC-2, and ABAS-II adaptive behavior profiles of 6- to 11-year-olds with HFASDs (within measure profile comparisons); (b) examine the extent to which these measures yielded similar findings on comparable scales (cross-measure comparisons); and (c) assess potential discrepancies between cognitive ability and adaptive behavior (as measured by the different adaptive behavior instruments).

## 2. Method

### 2.1. Participants

The sample for this study consisted of 50 children, ages 6 to 11 years (M = 9.58, SD = 1.41), with HFASDs. The children were participating in separate psychosocial intervention studies for children with HFASDs, and all met inclusion criteria using a multiple-gate screening procedure. Criteria included a prior clinical diagnosis of an ASD, Wechsler Intelligence Scale for Children-4th Edition (WISC-IV; [21]) short form IQ > 70, and a receptive or expressive language score ≥80 on a short-form of the Comprehensive Assessment of Spoken Language (CASL; [22]). In addition, diagnostic status was confirmed using the Autism Diagnostic Interview-Revised (ADI-R; [23]; completed by parents). 

In the first gate, parents submitted documentation of a prior diagnosis of Asperger's, autism, or PDD-NOS made by a licensed psychologist or psychiatrist and all relevant clinical and educational reports and records. Parents also completed a demographic form and developmental history. Once the required documents were received, the case was transferred to the second gate where two members of the senior research team independently reviewed the case using a standardized checklist composed of items indicating cognitive ability, current language levels, and DSM-IV-TR criteria (i.e., social interaction impairments and restricted, repetitive, and stereotyped patterns of behaviors or interests; [1]). Each made an independent determination as to whether the documents supported the presence of a HFASD and clinical consensus between the two senior researchers was necessary to move the case to the third gate. In the third gate, children completed an evaluation which included cognitive (WISC-IV [21] short form) and language (CASL [22] short-form) testing, and parents were administered the ADI-R [23]. Upon completion, the two senior researchers reviewed the evaluation results and prior reports using the standardized checklist and independently made a determination as to whether results were consistent with a HFASD and met inclusion criteria. Consensus between the two researchers was required for inclusion.

The sample for the current study was predominantly male (94%) and Caucasian (90%), with an average WISC-IV short-form IQ of 104.15 (SD = 13.55). The mean expressive and receptive language scores of the sample fell in the average range (M = 102.98 (SD = 15.44) and M = 108.54 (SD = 13.72), resp.). Average reported parent education was 15.43 (SD = 2.10) years. A detailed description of the sample characteristics is presented in Table 1.

### 2.2. Instrumentation

#### 2.2.1. Wechsler Intelligence Scale for Children-4th Edition (WISC-IV)

Cognitive ability was evaluated using a 4-subtest short form of the WISC-IV [21] consisting of the Block Design, Similarities, Vocabulary, and Matrix Reasoning subtests. Methods provided by Tellegen and Briggs [24] were used to calculate short-form reliability and validity coefficients and the deviation quotient formula, based on standardization information in the WISC-IV technical manual. The short-form composite yielded an internal consistency estimate of .95 and correlated .92 with the Full Scale IQ.

#### 2.2.2. Comprehensive Assessment of Spoken Language (CASL)

A 4-subtest short form of the CASL [22] was used as a screening measure for expressive and receptive language skills. The expressive language composite consisted of the Antonyms and Syntax Construction subtests and yielded internal consistency estimates ranging from .89 to .93. The receptive language composite consisted of the Synonyms and Paragraph Comprehension subtests for those ≥7 years (or Synonyms and Sentence Completion subtests for 6 year olds), and yielded internal consistency estimates ranging from .85 to .90. Composite internal consistency reliabilities and deviation quotients were calculated using the formulas provided by Tellegen and Briggs [24].

#### 2.2.3. Autism Diagnostic Interview-Revised (ADI-R)

The ADI-R [23] is a 93-item standardized diagnostic interview administered to a caregiver familiar with the developmental history and current behavior of the person being evaluated. The interview focuses on three domains (i.e., Reciprocal Social Interaction, Communication, and Restricted, Repetitive, and Stereotyped Behavior). Interrater reliability for a sample of individuals between 5 and 29 years was approximately 0.80. Validity evidence indicates that the ADI-R effectively discriminates between ASD and non-ASD samples [23]. 

#### 2.2.4. Vineland Adaptive Behavior Scales-Second Edition (VABS-II), Parent/Caregiver Rating Form

The VABS-II Parent/Caregiver Rating Form [15] assesses adaptive behaviors of individuals from birth through 90 years, 11 months. For the age range covered by this study, the VABS-II yields nine subdomain scores (i.e., Expressive, Receptive, Written, Personal, Domestic, Community, Interpersonal, Play and Leisure, and Coping), three domain scores (i.e., Communication, Socialization, and Daily Living Skills) each comprised of three subdomains, and an overall Adaptive Behavior Composite score. (An optional Maladaptive Behavior domain is available, as well as a Motor Skills domain for children younger than 7 years. However, neither of these was used in the current study.) Items are arranged in a developmental sequence within each subdomain, with each item rated on a 3-point scale (i.e., 0 = Behavior Never Performed, 1 = Behavior Sometimes or Partially Performed, or 2 = Behavior Usually or Habitually Performed). 

 The VABS-II Adaptive Behavior Composite and domains yield age-based standard scores with a normative M = 100 and SD = 15, and the subdomains yield standard scores with a M = 15 and SD = 3. Reliability and validity estimates reported in the test manual are based on pooled data from both the interview and parent/caregiver rating forms [15]. For the age range covered in this study, internal consistency reliability estimates range from .95 to .97 (Mdn = .97) for the Adaptive Behavior Composite, .89 to .94 (Mdn = .93) for the Communication domain, .88 to .93 (Mdn = .92) for the Daily Living Skills domain, and .89 to .95 (Mdn = .93) for the Socialization domain. Median split-half reliability estimates across the nine VABS-II subdomains for ages 6 to 11 years range from .74 to .89. Concurrent validity of the VABS-II is supported by moderate correlations with other scales assessing adaptive performance. Adjusted correlations, reported in the VABS-II manual for participants age 5 to 20 years, between the VABS-II domains/composite and equivalent ABAS-II domains/composite, were moderate to high (i.e., *r* = .60 to .74 for the domain scores and .78 for the overall adaptive composites). Correlations between the VABS-II adaptive domains/composite and the BASC-2 PRS-C adaptive scales were generally moderate and range from .38 to .60 [15]. 

#### 2.2.5. Behavior Assessment System for Children-Second Edition, Parent Rating Scales (BASC-2 PRS)

The BASC-2 PRS [19] is a multidimensional assessment system that evaluates both clinical and adaptive aspects of behavior and personality. It was developed to assist in differential diagnosis for a range of DSM-IV-TR disorders, as well as treatment planning. While the BASC-2 also includes rating scales that can be completed by the child and/or teacher, only the Parent Rating Scale (PRS) was used in the present study and will be described here. The PRS provides information on the child's problem and adaptive behavior at home and in the community, and it is available for three age ranges including preschool (ages 2 to 5 years), child (6 to 11 years), and adolescent (12 to 21 years). Only the child form was used in the current study. Each item is rated on a 4-point frequency scale (i.e., 0 = Never, 1 = Sometimes, 2 = Often, and 3 = Almost Always), and item raw scores are summed and converted into standardized *T* scores (M = 50, SD = 10) for interpretation. 

Individual items measuring similar constructs/domains are grouped to form nine clinical behavior scales and five adaptive behavior scales. For the purposes of this study, only the adaptive scales (Adaptability, Social Skills, Leadership, Activities of Daily Living, and Functional Communication) and overall Adaptive Skills Composite were examined. For ages 6 to 11 years, coefficient alphas of the adaptive scales ranged from .73 to .87 (Mdn = .83). Adjusted test-retest reliabilities for the adaptive scales ranged from .82 to .84 (Mdn = .83) and was .90 for the Adaptive Skills Composite. Concurrent validity is supported in moderate correlations with other scales measuring similar skills/constructs. For additional psychometric details, see Reynolds and Kamphaus [19]. 

#### 2.2.6. Adaptive Behavior Assessment System-Second Edition (ABAS-II) Parent Form (Ages 5–21)

The ABAS-II Parent Form (Ages 5–21; [17]) is a comprehensive measure of adaptive functioning in the home and community. Each item is rated on a scale from 0 = Is Not Able to 3 = Always/Almost Always. The Parent Form (Ages 5–21) includes 10 skill areas (Communication, Community Use, Functional Academics, Home Living, Health and Safety, Leisure, Self-Care, Self-Direction, Social, and Work (Work is only for individuals ≥17 years)), which are combined to form three specific domain composites named Conceptual (CON; consisting of Communication, Functional Academics, Self-Direction), Social (SOC; combining Leisure and Social), and Practical (PRAC; encompassing Self-Care, Home Living, Community Use, and Health and Safety), and an overall General Adaptive Composite (GAC). Domain and composite scores have a norm-referenced M = 100 and SD = 15, and skill area scores have a norm-referenced M = 10 and SD = 3. Average internal consistency reliability estimates for 6- to 11-year-olds ranged from .94 to .99 for the domains/composite and .79 to .94 for the skill areas. For the 5 to 12 age range, corrected test-retest reliabilities ranged from .79 to .94 for all skill area scores, domains, and the GAC. Concurrent validity is supported by moderate correlations with other measures of adaptive functioning. Of relevance to the current study, correlations between the logically equivalent domains and composite scores of the ABAS-II and original VABS Interview Edition ranged from .49 to .70 [17]. No data on the relationship between the ABAS-II and BASC adaptive scales (for parents) or the ABAS-II and VABS-II Parent/Caregiver Rating or Interview Forms were available. See Harrison and Oakland [17] for additional psychometric details.

## 3. Procedures

This study was approved by the Institutional Review Board and conducted in compliance with the approved protocol. As previously noted all the children with HFASDs were screened using a structured protocol and met clearly defined inclusion criteria. Once a child was determined to have met inclusion criteria and prior to participation in an intervention, parents completed the ABAS-II, VABS-II Parent/Caregiver Rating Form, and BASC-2 PRS. The three rating scales were administered in counter-balanced order across participants to control for potential order effects.

Each adaptive behavior rating scale was immediately reviewed upon return in order to identify any omitted items and/or items with multiple responses. Errors were immediately addressed with parents and corrected. Each BASC-2 PRS protocol was scored using the BASC-2 ASSIST Plus computer scoring program, and each VABS-II protocol was scored using the VABS-II ASSIST computer scoring program. The ABAS-II protocols were hand scored in accordance with the manual. To ensure accuracy in scoring, each protocol was scored independently by two research assistants, with any discrepancies resolved by a third independent scorer. Using a similar protocol, scores from all measures were entered into a database and independently checked by a second member of the research team, with discrepancies resolved by a third team member. 

## 4. Results

Data for all three adaptive behavior measures were examined using three sets of analyses. The first examined score profiles within each instrument, the second examined mean differences between comparable scores across the three instruments, and the third assessed potential IQ versus adaptive behavior discrepancies for all three instruments. The overall, experiment-wise alpha level was kept at .05 using a Bonferroni-corrected, per-comparison alpha level of .001 (two-tailed) across all three sets of analyses. 

### 4.1. Within-Measure Profile Comparisons

Means and standard deviations for each subscale and composite by instrument are listed in Table 2. All scores in Table 2 are reported in their original metric, and they document the adaptive behavior profile of the sample for each instrument. 

#### 4.1.1. VABS-II

Using dependent samples *t*-tests, each of the nine subdomain scores of the VABS-II was compared to the average of the other eight remaining subdomain scores for statistical significance. This analytic method is consistent with the ipsative approach used for profile analysis in clinical practice, and it reduced the number of required subdomain comparisons to nine. Results indicated that the Interpersonal, Play and Leisure, and Receptive subdomains were significantly lower (*P* < .001), while the Written, Community, and Personal subdomains were significantly higher (*P* < .001) than the respective averages of the other subdomains. The three remaining subdomains were not significantly different (*P* > .05 in each case).

The three adaptive domain scores (i.e., Communication, Daily Living Skills, and Socialization) were compared to each other via one-way within-subjects ANOVA. It was predicted that the Socialization domain score would be significantly lower than the other two domain scores. The omnibus *F*-test was significant (*F*[2,  48] = 34.32, *P* < .001). Bonferroni-corrected post-hoc comparisons indicated that the Socialization domain was significantly lower than both the Communication and Daily Living Skills domains (*P* < .001), while the Communication and Daily Living Skills domains were not significantly different from each other (*P* = .364).

#### 4.1.2. BASC-2 PRS

Potential differences among the five adaptive scales of the BASC-2 PRS were examined by comparing each scale's score to the average of the other four scales' scores for statistical significance using dependent samples *t*-tests. This strategy reduced the number of required comparisons to five. All comparisons for the BASC-2 PRS adaptive scales were nonsignificant (*P* > .001). (This result was the same whether the Bonferroni-corrected alpha of .001 was used or an uncorrected alpha of .05.) 

#### 4.1.3. ABAS-II

The nine skill area subscales of the ABAS-II were each compared to the average of the other eight skill area subscale scores for statistical significance using dependent samples *t*-tests. This reduced the number of required comparisons to nine. Results of the comparisons indicated that the Social, Home Living, and Self-Direction scores were significantly lower (*P* < .001), while the Functional Academics and Health and Safety scores were significantly higher (*P* < .001) than the respective averages of the other subscales. 

The three adaptive domain scores (i.e., Conceptual, Practical, and Social) were each compared to each other using one-way within-subjects ANOVA. It was predicted that the Social domain score would be significantly lower than the other two domain scores. The overall omnibus *F* test was significant (*F*[2,  48] = 18.92, *P* < .001). Bonferroni-corrected post-hoc comparisons indicated that the Social domain score was significantly lower than the Conceptual domain score (*P* < .001), while the Practical domain score did not differ significantly from either the Conceptual (*P* = .083) or Social domain (*P* = .037) scores following the alpha correction. 

### 4.2. Cross-Measure Comparisons

The cross-measure comparisons are presented in Table 3. To make these comparisons possible, the scores from all three adaptive behavior measures were converted into a common metric (a deviation quotient metric with a normative M = 100 and normative SD = 15). This allowed for direct score comparisons across the different measures. The most logically equivalent adaptive scores were compared across each of the three measures. In some cases, such scores were only available across two measures (i.e., VABS-II versus ABAS-II). Comparisons were made across the instruments using one-way within-subjects ANOVAs, at a per-comparison alpha level of .001. 

For all 12 comparisons, the overall *F* test for each ANOVA was significant at *P* < .001. In cases where scores from all three instruments were compared, a significant omnibus *F* was followed by post-hoc comparisons (see Table 3). 

Five of the 12 cross-measures comparisons involved all three instruments. When such comparisons were made using the most logical estimates of overall adaptive behavior, communication/conceptual skills, coping/adaptation skills, and daily living/practical skills, the VABS-II was found to yield significantly higher scores than both the BASC-2 PRS and ABAS-II (*P* < .001), while the BASC-2 PRS and ABAS-II were not significantly different from each other (*P* > .05). The remaining comparison across the three measures involved the social functioning construct. In this case, post-hoc comparisons revealed that the BASC-2 PRS was significantly higher than the ABAS-II (*P* < .001), but that the VABS-II was not significantly different from either the BASC-2 PRS (*P* = .028) or ABAS-II (*P* = .026) using the Bonferroni-corrected .001 alpha. 

Because the BASC-2 PRS had fewer adaptive scales than the other two instruments, the remaining analyses involved comparisons only between the VABS-II and ABAS-II. For six out of seven of the two-test comparisons, the VABS-II score was significantly higher than the ABAS-II score. The one exception was the Play and Leisure comparison, which showed the opposite outcome.

### 4.3. IQ versus Adaptive Behavior Discrepancies

Mean differences between the WISC-IV short-form IQ score and each of the nine adaptive behavior summary scores (i.e., one composite from the BASC-2 PRS, one composite and three domain scores from the VABS-II, and one composite and three domain scores from the ABAS-II) in deviation quotient scaling were assessed using dependent samples *t*-tests. Means for all nine adaptive behavior composites and domain scores (Table 4) were significantly lower (*P* < .001) than the mean short-form IQ (M = 104.15). Deviation quotient discrepancies ranged from 12.71 points (VABS-II Daily Living Skills domain) to 32.07 points (ABAS-II Social domain; see Table 4). 

## 5. Discussion

Assessment of adaptive functioning is considered one of the most critical components of comprehensive evaluations for children with HFASDs as it captures information on the child's functional adjustment in everyday situations [7]. This study was conducted to provide additional data on the adaptive behavior profiles of children with HFASDs using the VABS-II, BASC-2, and ABAS-II, cross-measure comparability, and the discrepancy between IQ and adaptive behaviors. The study also addressed the need for adaptive behavior studies that use a more narrowly defined and homogeneous sample (i.e., children with HFASDs).

Profiles for the three measures are detailed in Table 2. Within the VABS-II, the Socialization domain score fell in the *Moderately Low* range [15] and was found to be significantly lower than the Communication and Daily Living Skills (DLS) domain scores, yet the Communication and DLS scores did not differ significantly. The finding of significantly impaired socialization adaptive skills is consistent with other studies of individuals with HFASDs; however, other studies have found DLS levels to be generally commensurate with the Socialization domain (e.g., Klin et al. [2]; Liss et al. [11]). For the Communication and DLS domains, the current sample had scores that were comparable and in the *Adequate* range [15]. The strength in communication was not necessarily surprising given the relative strength in language for children with HFASDs. Although other studies using the original version of the VABS with children and/or adolescents with HFASDs have also found Communication to be the highest of the domain scores, their Communication scores tended to fall substantially below the average range (e.g., Klin et al. [2]; Liss et al. [11]; Saulnier and Klin [14]). This is also true for the DLS domain (when reported) in those studies (i.e., DLS substantially below the average range). Some of the discrepancies in findings between studies may be due to differences in the ages of the samples, inclusion criteria, and so forth, or use of the VABS-II in this study. Beyond the domain scores, comparisons of the VABS-II subdomain scores revealed relative strengths in the Written, Community, and Personal areas and weaknesses in the Interpersonal, Play and Leisure, and Receptive areas. The extent to which these subdomain scores were consistent with other studies could not be determined as other studies using the VABS with individuals with HFASDs have not reported the subdomain scores. 

Within the BASC-2, scores were generated for the five adaptive scales and an overall composite (the BASC-2 does not contain separate adaptive domains). Results of the scale comparisons were all non-significant and reflected a similar level of adaptive impairment. Scores across the Adaptability, Social Skills, Leadership, Activities of Daily Living, and Functional Communication scales fell in the *At-Risk* range [19]. Although the lack of differentiation of the Social Skills scale (as a relative weakness) from the other adaptive scales might appear unusual, the finding is consistent with Volker et al. [18] who also found that all five of the BASC-2 adaptive scales were in the at-risk range for a sample of 6- to 16-year-olds with HFASDs. 

For the ABAS-II, the Conceptual domain score fell in the *Below Average* range (80 to 89), and the Practical and Social domain scores were in the *Borderline* range (71 to 79) [17]. The Social domain score was found to be significantly lower than the Conceptual domain score. This discrepancy was expected given the characteristic social impairments (reflected in the Social domain) and relative language and academic strengths (captured in the Conceptual domain) of children with HFASDs. The Practical domain score did not differ from the Conceptual or Social domain scores. While the Social domain score was significantly lower than the Conceptual score, the Practical domain score fell in between the Social and Conceptual scores. This resulted in non-significant differences between the daily living type skills captured by the Practical domain score and the other domain scores. The discrepancy between the Social domain and Conceptual domain scores in this study is similar to that reported by Kenworthy et al. [16] for 12- to 22-year-olds with HFASDs. In contrast, Kenworthy et al. [16] found the Practical domain was similarly impaired as the Social domain (M = 73 for both domains). Although the reason for the somewhat different findings for the Practical domain is unknown, it is possible that basic and instrumental daily living skill deficits (Practical domain) become more pronounced during adolescence and young adulthood for individuals with HFASDs. Analyses of the skill area scores indicated relative strengths in Functional Academics and Health and Safety and relative weaknesses in Social, Home Living, and Self-Direction. Kenworthy et al. [16] also identified the Functional Academics skill area as a strength and Social as a weakness for their sample.

The second aim of this study was to examine the comparability of the measures on composites and scales measuring similar adaptive constructs and skills. Overall, the VABS-II yielded scores that were significantly higher on 10 of the 12 comparisons. On four of the five comparisons that encompassed all three measures (i.e., overall adaptive skills, communication/conceptual, coping/adaptation, and daily living/practical skills), the VABS-II yielded significantly higher scores than both the BASC-2 and ABAS-II, while the BASC-2 and ABAS-II did not differ significantly on these skills. On the Social domain, which is critically important for children with HFASDs, the BASC-2 score was significantly higher than the ABAS-II, and the VABS-II did not differ significantly from either the ABAS-II or BASC-2. Based on the common metric, the VABS-II Socialization domain score fell approximately midway between the ABAS-II and BASC-2 (i.e., BASC-2 M = 81, VABS-II M = 76, and ABAS-II M = 72). 

Seven comparisons only involved the VABS-II and ABAS-II (due to the BASC-2 having fewer adaptive scales). On these comparisons of similar skill subdomains/areas, the VABS-II yielded significantly higher scores for six of the seven comparisons. The only subdomain/skill area which yielded a contradictory finding was Play and Leisure which was significantly lower for the VABS-II compared to the ABAS-II. 

Overall, these findings indicated that the degree of impairment in adaptive levels varied considerably across the instruments. In general, the VABS-II yielded higher scores than the ABAS-II. The two measures did not differ significantly on the social domain and both captured significant adaptive social deficits which is especially important for HFASDs; however, their mean scores differed by four points. The potential impact of the significantly higher scores yielded by the VABS-II, as well as the non-significant 4-point discrepancy, on eligibility determinations, requires additional investigation beyond this study. The BASC-2 yielded adaptive scores that were largely similar to the ABAS-II, with one notable exception which involved adaptive social competence. For this comparison, the BASC-2 was significantly higher than the ABAS-II which may be particularly problematic for the population of children with HFASDs who are defined by social impairment. Conclusions about the comparability of the BASC-2 with the ABAS-II or VABS-II should be viewed with caution as the BASC-2 is a broader measure of clinical and adaptive functioning, and it contains far fewer adaptive skills scales for comparisons. 

The third aim of the study was to examine the discrepancy between IQ and the adaptive behavior measures for this sample of children with HFASDs. Results indicated that all adaptive domain scores and composites fell significantly below the mean IQ of the sample. Examination of the magnitudes revealed that the largest discrepancies occurred for the social domains on the VABS-II and ABAS-II. These results are generally consistent with other studies using the original VABS and the ABAS-II (e.g., Klin et al. [2]; Lopata et al. [25]). Although the BASC-2 only provides a social skills scale (no social domain), the overall adaptive composite showed a discrepancy with IQ that was only slightly smaller in magnitude than the Socialization domain of the VABS-II. It is unknown whether this may suggest that the overall adaptive skills composite is tapping primarily social aspects of adaptive functioning, or whether the individual adaptive scales of the BASC-2 are not subskill specific and instead are each tapping a more broad set of adaptive skills. Overall, results of this study support prior findings indicating a significant gap between cognitive ability and adaptive skills in this population [14]. Klin and Volkmar [9] characterized this as “a significant discrepancy between cognitive potential (i.e., IQ) (of these children) and their ability to translate this into adaptive functioning” (page 346). 

This study had a number of strengths. First, a homogenous sample of children with HFASDs was utilized which is uncommon in the adaptive behavior research as many studies have had samples of both lower- and higher-functioning individuals with ASDs [5, 10, 11]. Additional strengths involved the well-characterized sample and rigorous screening procedures which included IQ and language testing and administration of the ADI-R. The sample size of 50, though not large for psychometric samples, is large for a carefully screened population of children with HFASDs. The magnitude of the effects allowed for considerable statistical power despite the number of comparisons and stringent alpha corrections used. This study was also the first to examine three different adaptive behavior measures that were completed by the same group of parent informants. Despite these strengths, the study had several weaknesses. Although stringent alpha corrections were used, a larger sample size would have been helpful given the number of required comparisons. The findings regarding the VABS-II are also limited to the rating scale version of the measure. Since the VABS-II is also available in an interview format, it is unknown whether similar findings would occur if administered via interview. Lastly, the cross-measure analyses were limited to mean score comparisons. Future studies should extend these to include correlational analyses of the scores. Given these limitations, study replications and extensions are needed of the adaptive functioning of children with HFASDs and the measures used to assess adaptive functioning. 

## 6. Conclusions

Assessment of adaptive functioning is critical for children with HFASDs. As such, clinicians require measures that accurately capture the adaptive strengths and weaknesses of these children. Rating scales represent an important assessment tool to gather such information as parents can complete the scales independently and quickly [16]. Parents are also considered a critical source of adaptive-functioning information as they observe the children in everyday naturalistic situations, as well as across different settings [7]. Results of this study suggested that the VABS-II and ABAS-II both captured the characteristic social impairments and relative academic strength of the children in the sample. The BASC-2 appeared to capture a similar level of adaptive impairment across its scales. Perhaps the most interesting finding was that the VABS-II tended to yield significantly higher scores than the other measures. This may be problematic for the VABS-II as it was designed specifically to assess adaptive behaviors. The findings suggest that researchers should be cautious in assuming that different adaptive behavior measures are comparable. The lack of cross-measure comparability may explain some of the discrepant findings across studies. Clinicians should be equally cautious when selecting an adaptive behavior measure as eligibility determinations may be affected by the measure used. The manner in which the determinations will be influenced may be affected by the scales used to make the eligibility decision. For example, clinicians using the overall adaptive composite score and/or the nonsocial adaptive scales to determine eligibility would likely find higher scores yielded by the VABS-II and fewer children meeting eligibility criteria. In contrast, if relying on the adaptive social scales, the BASC-2 would appear to be most likely to yield fewer positive eligibility determinations. Additional studies of the impact of scale selection on eligibility decisions are clearly needed and should be designed to specifically answer such questions; however, the ABAS-II may provide some additional sensitivity for lower-level adaptive skills which appear to characterize many children with HFASDs. Lastly, children in the study demonstrated significant discrepancies between their IQ and adaptive functioning, with the greatest discrepancies occurring on the social domains of the VABS-II and ABAS-II. 

## Figures and Tables

**Table 1:** Sample demographic characteristics.

Characteristic	Participants (*N* = 50)
	M (SD)

Age in years	9.58 (1.41)
Parent education in years	15.43 (2.10)
WISC-IV	
Short-form IQ	104.15 (13.55)
CASL	
Short-form expressive language	102.98 (15.44)
Short-form receptive language	108.54 (13.72)

	*n* (% of total)

ASD diagnosis	
Asperger's disorder	35 (70.0%)
PDD-NOS	11 (22.0%)
Autistic disorder	4 (8.0%)
Gender	
Male	47 (94.0%)
Female	3 (6.0%)
Ethnicity	
Caucasian	45 (90.0%)
African-American	1 (2.0%)
Hispanic	1 (2.0%)
Asian-American	2 (4.0%)
Other	1 (2.0%)

Diagnostic category data reported in this table constitute diagnoses made by external clinicians (i.e., contained in reports submitted by parents). All testing to determine inclusion in this study (i.e., WISC-IV, CASL, and ADI-R) was done by members of the research team. WISC-IV: Wechsler Intelligence Scale for Children-4th Edition; CASL: Comprehensive Assessment of Spoken Language; PDD-NOS: pervasive developmental disorder not otherwise specified.

**Table 2:** Means and standard deviations in the original metrics for the VABS-II, BASC-2 PRS, and ABAS-II.

VABS-II		BASC-2		ABAS-II	
Subdomain	M (SD)	Scale	M (SD)	Skill Area	M (SD)

Expressive	12.50 (2.61)	Adaptability	36.46 (9.29)	Communication	5.88 (2.57)
Receptive	11.26 (2.31)	Social Skills	37.48 (8.21)	Community Use	6.70 (3.63)
Written	15.12 (3.14)	Leadership	37.94 (7.47)	Functional Academics	8.48 (2.57)
Personal	14.06 (3.62)	Activities of Daily Living	37.52 (9.11)	Home Living	4.42 (3.02)
Domestic	12.30 (2.53)	Functional Communication	36.84 (8.63)	Health and Safety	7.26 (2.95)
Community	14.36 (2.99)			Leisure	5.90 (2.36)
Interpersonal	9.78 (2.84)			Self-Care	6.36 (2.87)
Play and Leisure	9.40 (2.84)			Self-Direction	4.78 (3.30)
Coping	12.54 (2.88)			Social	3.12 (2.75)

Domain/composite		Composite		Domain/composite	

Communication	88.38 (12.20)			Conceptual	81.26 (12.10)
Daily Living Skills	91.44 (14.65)			Practical	77.56 (16.75)
Socialization	76.12 (13.39)			Social	72.08 (11.20)
Adaptive Behavior Composite	83.52 (11.05)	Adaptive Skills Composite	34.84 (7.82)	General Adaptive Composite	73.64 (12.99)

For the VABS-II, normative subdomain score M = 15 and SD = 3 and normative domain score and composite M = 100 and SD = 15. For the BASC-2 PRS, normative M = 50 and SD = 10 for all scores. For the ABAS-II, normative skill area score M = 10 and SD = 3 and normative domain score and composite M = 100 and SD = 15.

**Table 3:** Cross-measure comparisons of the VABS-II, BASC-2 PRS, and ABAS-II scores assessing similar constructs.

VABS-II		BASC-2		ABAS-II		Within-row statistical results
Domain/subdomain/composite	M (SD)	Scale/composite	M (SD)	Domain/skill area/composite	M (SD)	
Communication domain	88.38 (12.20)	Functional Communication	80.26 (12.94)	Conceptual Domain	81.26 (12.10)	*F*(2, 48)* = *14.59*, P <*.001 Post hoc: V > (B = A)
Expressive	87.26 (12.77)			Communication	79.40 (12.84)	*F*(1, 49)* = *15.69*, P <*.001 Direction: V > A
Receptive	81.79 (11.89)					
Written	100.75 (16.18)			Functional Academics	92.40 (12.87)	*F*(1, 49)* = *24.27*, P <*.001 Direction: V > A
Socialization domain	76.12 (13.39)	Social Skills	81.22 (12.31)	Social Domain	72.08 (11.20)	*F*(2, 48)* = *14.81*, P <*.001 Post hoc: B > A; (V = B; V = A)
Interpersonal	73.87 (13.99)			Social	65.60 (13.73)	*F*(1, 49)* = *16.26*, P <*.001 Direction: V > A
Play and Leisure	71.51 (14.16)			Leisure	79.50 (11.79)	*F*(1, 49)* = *14.80*, P <.*001 Direction: V < A
Coping	88.02 (14.78)	Adaptability	79.69 (13.93)	Self-Direction	73.90 (16.48)	*F*(2, 48)* = *19.07*, P <*.001 Post hoc: V >(B = A)
		Leadership	81.91 (11.22)			
Daily Living Skills domain	91.44 (14.65)	Activities of Daily Living	81.28 (13.67)	Practical Domain	77.56 (16.75)	*F*(2, 48)* = *17.43*, P <.*001 Post hoc: V > (B* = *A)
Personal	95.38 (17.67)			Self-Care	81.80 (14.38)	*F*(1, 49)* = *38.95*, P <.*001 Direction: V > A
Domestic	86.51 (12.43)			Home Living	72.10 (15.09)	*F*(1, 49)* = *43.49*, P <*.001 Direction: V > A
Community	96.79 (14.78)			Community Use	83.50 (18.16)	*F*(1, 49)* = *21.11*, P <.*001 Direction: V > A
				Health and Safety	86.30 (14.77)	
Adaptive Behavior Composite	83.52 (11.05)	Adaptive Skills Composite	77.26 (11.73)	General Adaptive Composite	73.64 (12.99)	*F*(2, 48) = 18.31, *P* < .001 Post hoc: V > (B = A)

All scores have been transformed to deviation quotient scaling with a normative M = 100 and SD^ ^= 15 in order to facilitate comparisons across instruments. Statistical results consist of the omnibus *F *test for that row, followed by Bonferroni-adjusted post-hoc comparisons in cases where more than two scores are compared or a notation of the direction of the difference when only two scores are compared. For the statistical results: V = VABS-II, B = BASC-2 PRS, and A = ABAS-II.

**Table 4:** Discrepancies between mean IQ and adaptive behavior domain scores and composite scores.

Domain/composite	Standard score (M = 100)	Discrepancy
VABS-II		
Communication	88.38	15.77*
Socialization	76.12	28.03*
Daily Living Skills	91.44	12.71*
Adaptive Behavior Composite	83.52	20.63*
BASC-2		
Adaptive Skills Composite	77.26^†^	26.89*
ABAS-II		
Conceptual	81.26	22.89*
Social	72.08	32.07*
Practical	77.56	26.59*
General Adaptive Composite	73.64	30.51*

^†^The composite for the BASC-2 was the converted score used to create the common metric for the cross-measure comparisons (Table 3). Discrepancies were based on the difference between the group's IQ M = 104.15 and the domain/composite score from the adaptive measures.

*P < .001.
